# The Effect of Drying/Re-Flooding on Trace Metal, As and Se Fluxes in a Treatment Wetland: Addressing Growing Environmental Concerns

**DOI:** 10.3390/biology11020188

**Published:** 2022-01-25

**Authors:** Drew J. Hansen, Alex J. Horne

**Affiliations:** 1 Agricultural and Environmental Chemistry Group, Department of Plant and Microbial Biology, University of California, Berkeley, CA 94720, USA; 2 Ecological Engineering Group, Department of Civil Engineering, University of California, Berkeley, CA 94720, USA; anywaters@comcast.net

**Keywords:** treatment wetland, sediment oxidation, re-flooding, trace metals, arsenic, selenium

## Abstract

**Simple Summary:**

The potential exposure of wildlife to toxic levels of metals following re-flooding in metal-contaminated water impoundments and coastal areas subject to sea level rise is of primary concern. Treatment wetlands are similar systems which enhance biogeochemical processes to remove low levels of pollutants including metals from wastewaters. Wetlands convert many dissolved metals to insoluble precipitates which are unavailable for biological uptake. When wetlands are dried/re-flooded, metals can be released. In this work, we present mass flux data for 11 metals, As and Se following drying/re-flooding in a constructed wetland used to treat oil refinery effluent. Following re-flooding, Co, Cr, Mg, Mn, Ni, S and Sr were continuously released to outflow, Ba, Cu, Fe, Mo and Zn showed zero net flux and As and Se were removed from inflow. We propose a mechanistic hypothesis consistent with the different flux patterns for metals which form sulfide precipitates. Our results suggest that following re-flooding, less-soluble sulfide metals may be immobilized prior to more-soluble metals in coastal systems and indicate that ponding strategies should be used to minimize metal pollution downstream. Research is urgently needed in these systems to improve metal removal efficiency, determine best management practices and for wildlife risk assessment.

**Abstract:**

The retention of heavy metals in water treatment wetlands is well documented, but little understood. Fluxes to and from sediments for moderate concentrations of dissolved metals are particularly unknown. Treatment wetlands are dried out seasonally or occasionally for maintenance. The extent to which heavy metals may be released by drying/re-flooding is of particular concern because of the potential for toxic levels of metals to be mobilized. A 36 ha treatment wetland receiving treated oil refinery effluent in California was dried for 6 months, then re-flooded to an average depth of >10 cm. The concentrations of 11 metals, As and Se in inflow, outflow, and porewaters were measured weekly for 4 months. Mass flux rates showed that the wetland acted as a sink for As and Se, six metals (Co, Cr, Mg, Mn, Ni, and Sr) and S were overall sources and five showed zero net flux (Ba, Cu, Fe, Mo, and Zn). Porewater results indicate that oxidation of the sediments caused the source metals to be released. Removal for As > Cu, Fe, Mo, Zn > Co, Mn, Ni was consistent with the thermodynamically-predicted ‘sulfide ladder’, suggesting that available sulfide was insufficient to re-sequester the entire pool of mobile chalcophile elements. Our results suggest that less-soluble sulfide metals may be immobilized prior to more-soluble metals following drying/re-flooding in coastal systems with multiple metal contaminants. Ponding for up to several weeks, depending on the metals of concern, will facilitate metal re-immobilization within sediments before waters are released and minimize impacts downstream. Research on how to speed-up the conversion of soluble metals to their insoluble sulfides or other immobilized forms is urgently needed.

## 1. Introduction

Increasing heavy metal pollution in soils and surface waters is a global concern [1,2]. Low-lying coastal areas, river flood plains, water retention basins and natural wetlands are particularly susceptible to metal accumulation from sources such as industrial activity, mining, acid rain, agricultural runoff and overbank flooding [2,3]. Drying and flooding cycles can mobilize metals from these systems [4,5], resulting in exposing wildlife to potentially toxic levels of metals as well as polluting discharges to the environment. Increasing our understanding of the processes which control metal mobility in these systems and which metals are more prone to mobilization are important areas of research today, as many of these systems are also subject to sea level rise and intensifying wet/dry cycles due to global warming.

In the United States, for flood control, conservation and the legal requirements for best management practices (BMP) to meet whole-basin plans depends on the increased use of detention basins which may be planted wetlands or vegetated by “self-design” plant growth. In either case, wet/dry cycles are the normal hydraulic regime. Sedimentation of particulate heavy metals is an attribute of such BMP basins. The USEPA regulates concentration and disposal of eight metals (Ag, As, B, Cd, Cr, Hg, Pb and Se). Metal removal by sedimentation is often 60–80% effective [6], depending on the type of BMP and water retention time. Maybeck [7], noted that sedimentation can reach 99%; however, “Such storage of contaminated sediment may last for decades to millennia, long after the cause of contamination has ceased. Many of these contaminated sites are still not registered. In some instances, the risks of their potential environmental impacts are not addressed (orphan pollution).” Increases in flooding events due to global warming add to the problem of metal releases from managed BMP sites [3] and the legacy impacts of orphan pollution.

Constructed treatment wetlands are increasingly being used to remove a wide variety of contaminants including metals from agricultural, municipal, and industrial wastewaters [8,9,10]. In recognition of their value as ecosystems and ability to control erosion, previously drained natural wetlands are being re-flooded in restoration efforts worldwide. Under anoxic conditions typical of wetlands and BMP basins many potentially toxic trace metals (e.g., Cu and Pb) become sequestered in sediments as sparingly-soluble metal sulfide precipitates, which are largely unavailable for biological uptake [11,12]. However, under oxic conditions sulfides become unstable and metals can be released. Many treatment wetlands are permanently flooded to maintain anoxia but occasionally must be dried out; operation on a seasonal (wet/dry) basis is also common. Because treatment wetlands typically contain low to moderate levels of metals, have controlled water flow and biogeochemical properties similar to BMP basins, they are ideal model systems in which to study the mobility of metals in response to drying/re-flooding. Unfortunately, the extent to which metals may be mobilized following drying/re-flooding has been the subject of few studies to date.

Under continuously-flooded conditions most dissolved metals accumulate in sediments with organic matter, clays and Fe(III) and Mn(IV) oxide minerals in the oxic and sub-oxic surficial layers of sediments (typically < 1.0 cm), and precipitate with dissolved sulfide in reducing porewaters. Indeed, metal sulfides are considered the main sink for most dissolved divalent cations in systems with anoxic sediments including wetlands, with iron sulfides (e.g., FeS and FeS_2_) the most abundant sulfide minerals by far in most systems [13]. The release of metals from sulfides following mild oxidative disturbance such as sediment suspension or the injection of oxic waters into sediments by burrowing animals has been the subject of many studies (e.g., [14,15,16,17]). Morse [18,19] showed that up to 90% of estuarine pyrite (FeS_2_) can oxidize and release the accompanying trace metals within 1 day of exposure to oxic seawater. It is thought that the oxidation of metal sulfides is a major source of dissolved metals in estuarine surface waters.

When sulfidic wetland sediments are completely dried out, much of the Fe(II) and Mn(II) released from the oxidation of pyrite and other metal sulfides (e.g., FeS, MnS) are retained as poorly-crystalline Fe(III) and Mn(IV) oxide precipitates, resulting in an increased abundance and downward distribution of easily-reducible oxide minerals throughout the upper sediments [20,21,22]. Fe(III) and Mn(IV) oxides are potent metal sorbents under oxidizing conditions and the rate at which they dissolve under reducing conditions can control metal release [23,24,25]. During short periods of inundation, this newly-formed sorbent pool can accumulate and stabilize the mobility of other trace metals, such as those which may have been released from oxidation [20,24,26,27]. However, when sediments are re-flooded and become reduced, Fe-reducing sediment zones are characterized by low sulfide levels [28,29]. Primarily, this is because sulfide rapidly reacts with Fe(III) oxides and dissolved Fe(II), which buffers the upward diffusion of sulfide produced in deeper sediments to low levels within the upper sediments [4,29,30]. Field studies have shown that persistent low-sulfide conditions due to Fe(III) oxide enrichment can influence the longer-term partitioning of trace metals in re-flooded wetland soils [20,28]. It seems likely then that the shorter-term release of metals following drying/re-flooding also occurs under low-sulfide conditions.

In a low-sulfate re-flooded freshwater system, Weber et al. [31] showed that when the supply of sulfide is limiting, multiple metals exhibit competitive precipitation behavior with the formation of less-soluble metal sulfides before those that are more soluble. We hypothesize that metals may be mobilized under sulfide limitation following drying/re-flooding in a high sulfate system and would exhibit similar behavior. To test this hypothesis, we evaluated the mass exchange between pore- and surface waters for multiple metals following drying/re-flooding of a Fe- and S-rich coastal constructed treatment wetland.

In the present study, we performed multi-element analysis on water samples from a 36 hectare (ha) treatment wetland used to polish oil refinery wastewater which were collected during a previous 16 week study [32]. The specific goals of the present work were: 1. Determine and characterize the extent to which different metals may be removed from, or released to, surface waters following drying/re-flooding with oil refinery wastewater. 2. Test the hypothesis that more-soluble chalcophile metals are mobilized to a greater extent than less-soluble metals following re-flooding in a coastal treatment wetland.

The water samples reported here were archived following a 1995 field study which focused on an industrial discharge that was locally important. We did not realize at the time that similar flooded enclosure wetlands (BMPs) would become the dominant method of all watershed pollution control. In the intervening 20 years, considerable measurements of retention of particulate-related pollutants in BMPs have been performed, but relatively little on soluble pollution, and almost none on dissolved trace metals [33]. Today, increases in extreme weather events (droughts and floods), global metal pollution and water conservation (e.g., stormwater impoundments) have intensified the urgency to develop management strategies and increase scientific understanding about metal mobility in re-flooded systems. Thus, we present these data in relation to more recent information and scientific research. We also note that in last 20 years, the high percentage of US rivers and streams subject to metal pollution (~60%) has not fallen, possibly because management of BMP wetlands, basins and retention ponds still remains more of an art than science. We hope this work will assist in developing management strategies (e.g., a 3–4 week holding period) for applicable BMP systems, encourage research and add to scientific understanding in these important areas of growing environmental concern.

## 2. Materials and Methods

### 2.1. Study Site

**Figure 1:**
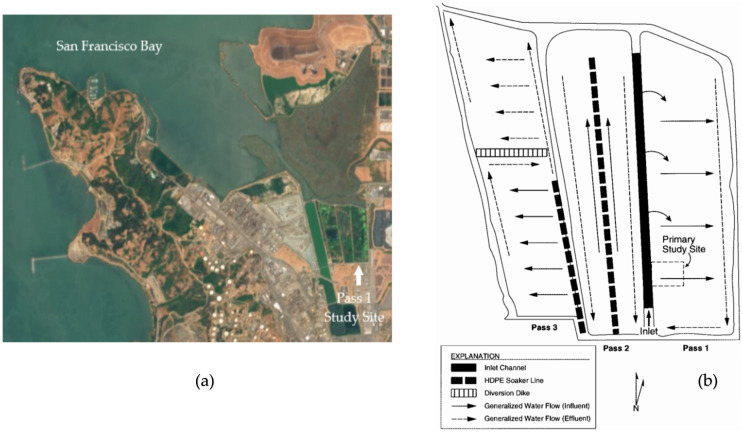
(**a**) The Chevron wetland in relation to San Francisco Bay; (**b**) schematic of the wetland showing generalized water flow and location of the primary study site in Pass 1.

The study site was a surface-flow constructed wetland situated near the mouth of San Pablo Bay, in San Francisco Bay (37.947° N; −122.382° W, Figure 1). The 36 ha wetland has been used to polish treated oil refinery wastewater since 1991, and is divided into three nearly equal sections (Passes 1–3). The work reported here concerns the first 12 ha section (Pass 1), because previous research showed that most metal removal occurred there (e.g., 75% of removal for total Se) [32,34].

Pass 1 was dried out and all above-ground organic matter was removed over six months in the winter and spring previous to the study. It was then re-flooded during the four weeks before the study began, and throughout the 16 week study period (26 June–15 October 1995), to an average depth > 10.0 cm. Water flow rates into and out of Pass 1 were monitored twice daily. The mean inlet and outlet flow rates were 6.4 ± 1.4 and 6.7 ± 3.0 million L day^−1^ (mean ± SD), respectively. The average residency time of water in Pass 1 was approximately 3 days. Following re-flooding, plant regrowth occurred rapidly, primarily dense stands of saltmarsh bulrush (*Scirpus maritimus* and *Scirpus robustus*) and cattails (*Typha angustafolia*, *Typha domingensis*, and *Typha latifolia*; B. Ertter, pers. obs.). A more detailed description of the entire wetland, flow rate determinations and mass flux calculations are reported in Hansen et al. [32].

### 2.2. Water Sample Collection and Analysis

Water samples were collected weekly from inlet and outlet waters and sediment porewaters using 10.0 cm Rhizon soil moisture samplers (pore size = 0.1 µm, Rhizosphere Research Products, Wageningen, The Netherlands) and 10.0 mL vaccutainers. Inlet and outlet waters were sampled with the samplers positioned horizontally, at a depth of 10.0 cm. Sediment porewater samples were collected to exclude the sediment water interface and surficial sediments by carefully inserting the samplers vertically, below any organic material to 1.0 cm below the surface of the sediments. Sediment porewater samples therefore represent composite conditions from 1.0 to 11.0 cm below the surface of the sediments. The sediment pore water samples reported here were collected in the primary study site (Figure 1). Water chemistry measurements for all water samples were made in the field immediately following collection. Sample pH was determined with a gel-filled combination electrode (Model 13-620-111, Fisher Scientific, Pittsburgh, PA, USA) calibrated with three buffers (pH = 4.0, 7.0, and 10.0); electrical conductivity (EC) was determined with a portable conductivity meter (Model CDH-80MS, Omega Engineering, Stamford, CT, USA) calibrated with three standards (1413, 2764, and 15,000 µS); and redox potential (Eh) was determined with a combination platinum-reference electrode (Model 13-620-82, Fisher Scientific, Pittsburgh, PA, USA) which was zeroed using Zoebell’s solution prior to each set of measurements. It should be noted that Eh measurements were made ex situ and water samples were briefly exposed to the atmosphere. Therefore, Eh results are qualitative and were likely higher than actual in situ conditions. To express Eh according to the standard hydrogen electrode (SHE), a value of 199 was added to recorded potentials. Water samples were brought back to the lab and stored at −80 °C until analysis.

Prior to analysis, samples were thawed, acidified (2–3% HNO_3_, A.C.S. grade), and gently vortexed several times. Total dissolved concentrations (0.1 µm filter size) were determined by ICP-AES (Model Iris HR, Thermo Jarrell Ash, Franklin, MA, USA) for the metals: Ba, Co, Cr, Cu, Fe, Mg, Mn, Mo, Ni, Sr, and Zn; the metalloid, As and S. Several field samples were used to determine background conditions and approximate concentrations for the 13 elements. Calibration standards were made within the appropriate range for each element. In addition, two separate multi-element quality control (QC) standards were analyzed with the field samples: QC#1 was used to make a dilution series (QC7A and QC2100, VHG Labs, Manchester, NH, USA); QC#2 was a trace-level check sample and was not diluted (QCTM-2 × 20, VHG Labs, Manchester, NH, USA). Total dissolved concentrations were determined for the nonmetal, Se, by atomic absorption spectroscopy as described in Hansen et al. [26]. Following sub-sampling for Se analysis, the water samples were stored at −80 °C for the subsequent ICP analysis reported here. Pb and Cd were either not present or were below detection limits for those elements. Porewater concentrations of total dissolved Fe and Mn were interpreted as qualitative indicators of dissolved Fe(II) and Mn(II) as most dissolved Fe occurs as Fe(II) [35,36,37].

Statistical analyses used the JMP IN statistical package, version 3.1.5 [38]. Statistically significant here is *p* < 0.05, unless indicated otherwise.

## 3. Results

### 3.1. Environmental Conditions

#### Water Chemistry

The 16 week mean values for pH, electrical conductivity (EC), and redox potential (Eh) measured in inlet, outlet, and porewaters are shown on Table 1.

Changes in pH, EC and Eh with time were also determined as follows:

Inlet and outlet pH significantly increased from 7.1 at the beginning, to 7.5 at the end of the study (*p* < 0.02). Porewater pH did not significantly change over time (remaining approximately 7.2) and became significantly lower than inlet and outlet values during the second half of the study (*p* < 0.01). The wetland sediments thus showed a pH buffering capacity, which is consistent with the findings of others that wetland sediments, in general, tend toward neutral pH [39].

Inlet, outlet, and porewater EC each significantly decreased during the study (*p* < 0.01), and inlet and outlet EC remained significantly less than porewater values over time (*p* < 0.02). The initial inlet, outlet, and porewater EC values were 4430, 4722, and 7600 µS cm^−1^, respectively. By week 16, inlet, outlet, and porewater EC decreased to 2683, 3542, and 5010 µS cm^−1^, respectively. Thus, as EC measures salt concentration, these data (see Supplementary Data S1) show an overall reduction in salinity occurred within the sediments during the study, and this reduction was most likely a function of the lower salt concentrations in surface (inlet and outlet) waters relative to porewaters, which resulted in leaching of mineral salts from porewaters.

Inlet, outlet, and sediment porewater Eh each significantly decreased during the study (*p* < 0.04). Initially, inlet, outlet, and sediment porewater Eh were similar (383, 356, and 381 mV, respectively). Porewater Eh then significantly decreased below inlet and outlet values over time (*p* < 0.02). The final values for inlet, outlet, and porewater Eh were: 221, 249, and 149 mV, respectively. As stated earlier, the wetland was kept dry for months prior to this study. Oxidizing conditions (>500 mV) presumably dominated the upper sediments during this time, as ground water levels fell to >15.0 cm below the sediment surface. These data show that the upper sediments became anoxic (<320 mV) soon after the beginning of the study, and that the upper sediments became progressively more reducing relative to surface waters over time.

### 3.2. Fe and Mn

**Figure 2:**
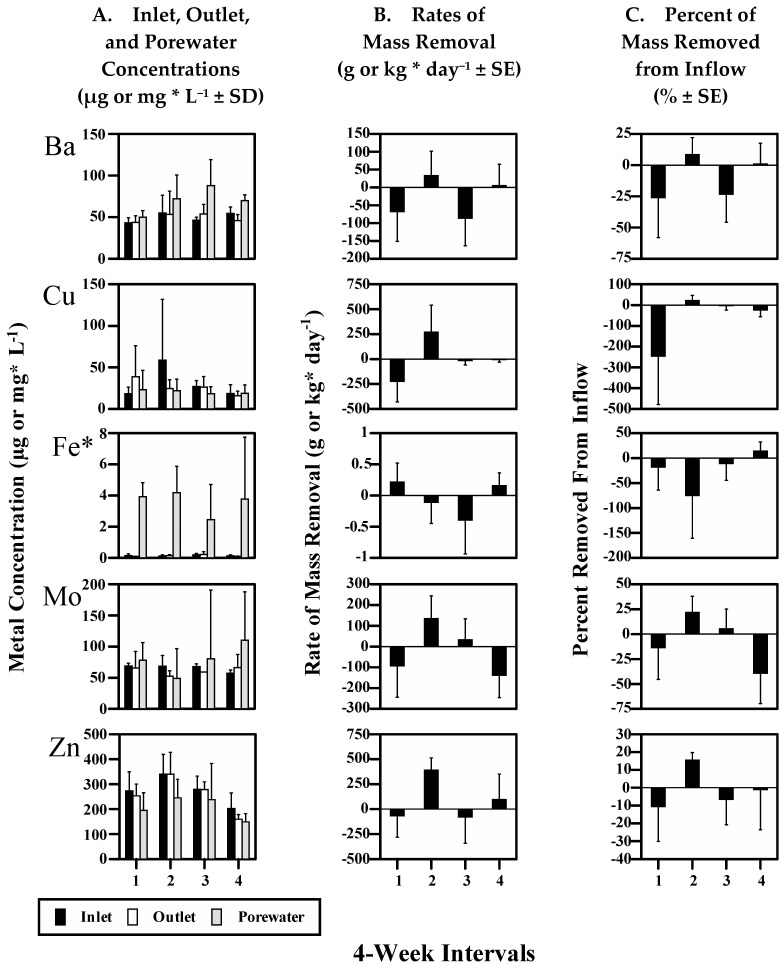
Zero-flux elements: Ba, Cu, Fe, Mo, and Zn. Periodic (4 week) mean values for: (**A**) Inlet, Outlet, and Porewater concentrations (µg or mg * L^−1^ ± standard deviation; *n* = 4 for inlet and outlet; *n* = 15–30 for porewater); (**B**) rates of mass removal (g or kg * day^−1^ ± standard error); and (**C**) percent of mass removed from inflow (±standard error) during the 16 week study period.

**Figure 3:**
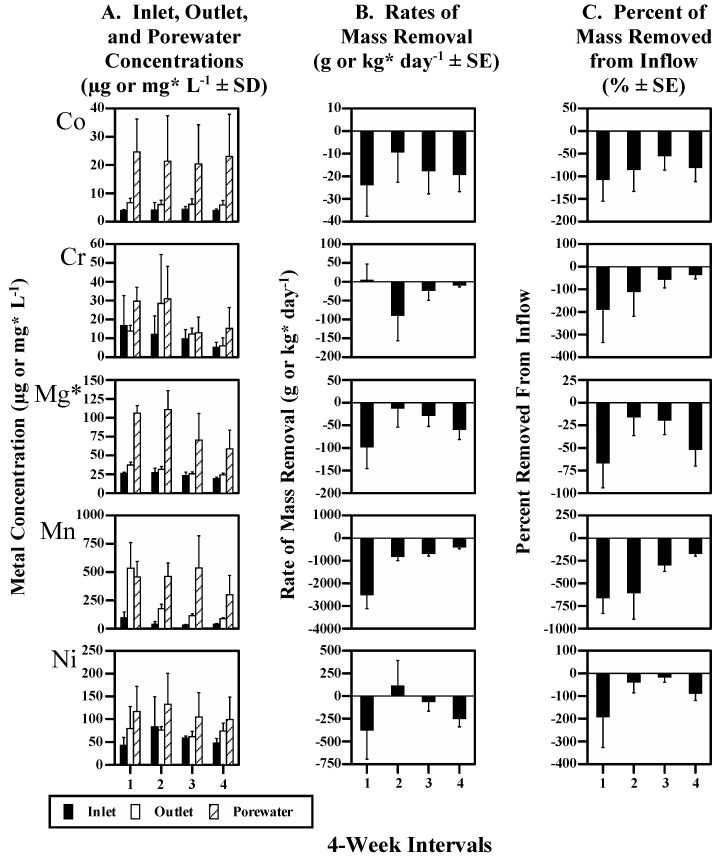

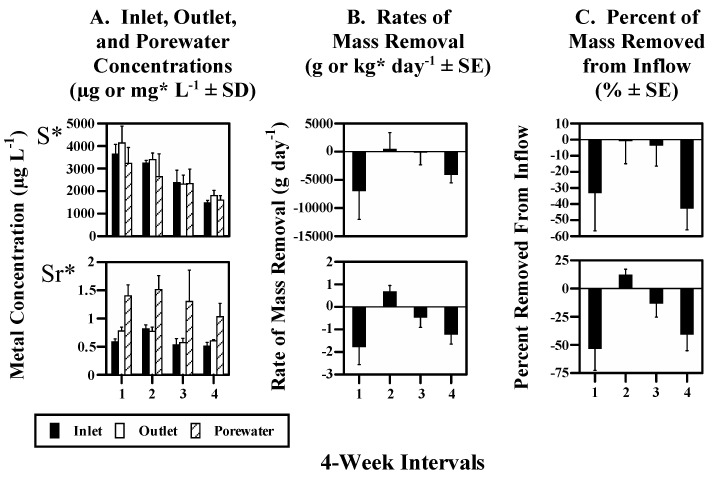
Source elements: S and Sr. Periodic (4-week) mean values for: (**A**) Inlet, Outlet, and Porewater concentrations (µg or mg * L^−1^ ± standard deviation; *n* = 4 for Inlet and Outlet; *n* = 15–30 for Porewater); (**B**) Rates of mass removal (g or kg * day^−1^ ± standard error); and (**C**) Percent of mass removed from inflow (±standard error) during the 16-week study period.

The concentrations of dissolved Fe and Mn in porewaters remained elevated above surface water concentrations throughout the study (Figure 2A and Figure 3A), which is consistent with the reductive dissolution of Fe(III) and Mn(IV) oxides [30,37,40]. Mn porewater concentrations decreased somewhat by the end of the study as Mn removal rates trended from negative toward positive values (Figure 3A–C), indicating that Mn was continuously released from sediments to surface waters and outflow. Fe porewater concentrations remained constant ca. 20 times higher than surface water concentrations. These data indicate that Mn(IV) reduction began to decrease by the end of the study and that Fe(III) reduction was sustained throughout the study within the upper sediments.

### 3.3. Element Concentrations in Porewaters and Surface Waters and Mass-Flux Rates

**Figure 4:**
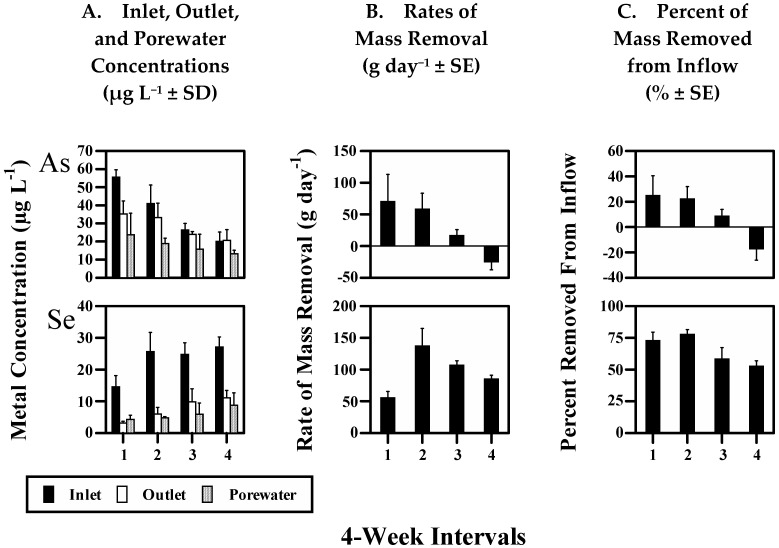
Sink elements: As and Se. Periodic (4 week) mean values for: (**A**) Inlet, Outlet, and Porewater concentrations (µg L^−1^ ± standard deviation; *n* = 4 for inlet and outlet; *n* = 15–30 for porewater); (**B**) rates of mass removal (g day^−1^ ± standard error); and (**C**) percent of mass removed from inflow (±standard error) during the 16 week study period.

The measured elements were grouped according to relative differences in overall (16 week) mean mass flux rates (Table 2). The overall removal rates were positive for ‘Sink’ elements (As and Se), negative for ‘Source’ elements (Co, Cr, Mg, Mn, Ni, S, and Sr), and were not different from zero for ‘Zero-flux’ elements (Ba, Cu, Mo, Zn, and Fe). Similarly, the periodic (4 week) mean removal rates show that mass removal was sustained over time for Sink elements (Figure 4B,C), occurred only during Period 2 for Zero-flux elements, except for Fe (Figure 2B,C), and was always negative for Source elements, except for Sr also during Period 2 (Figure 3B,C). Thus, these data show that during the course of the re-flooding study, the wetland removed Sink elements from inflow, released Source elements to outflow, and that periods of removal from inflow were equally offset by release to outflow for Zero-flux elements.

Porewater concentrations were compared to inlet and outlet concentrations with similar results. Sink element overall mean porewater concentrations were significantly less than inlet concentrations (Table 2), and were generally less than inlet and outlet concentrations over time (Figure 4A). In contrast, Zero-flux element mean porewater concentrations were not significantly different from inlet and outlet concentrations (Table 2), and were similar to inlet and outlet concentrations over time (Figure 2A), except for Fe. In sharp contrast, Source element mean porewater concentrations were significantly greater than inlet and most outlet concentrations (Table 2), and were generally greater than inlet and outlet concentrations over time (Figure 3A), except for S. The partitioning of dissolved compounds between surface waters and sediments is primarily diffusive, driven by differences in concentration between the two phases with a strong gradient occurring in sediment porewaters. Thus, as the relative differences between porewater and surface (inlet and outlet) water concentrations are similar to the differences based on overall mass fluxes (above), these results indicate that the observed positive and negative overall mass fluxes for Sink and Source elements occurred between surface waters and sediments. Similarly, these results indicate that the overall exchange between surface waters and sediments for Zero-flux elements was limited.

## 4. Discussion

### 4.1. Metal Mobilization following Drying/Re-Flooding

The concentrations of heavy metals measured here are similar to many found in wastewaters or urban runoff, though Se is higher in dryer climates and for some oil and coal wastes. They are much lower than from most acid metal mines but still much higher than is safe for sensitive organisms such as oysters or young trout or salmon. Metal toxicity is often dependent on the ionic state of the metal, or if it is chelated, but this is a complex process and can change easily. For released metals following dry/wet cycles it is likely that the more toxic forms will predominate, at least initially.

Following re-flooding of this treatment wetland, most metals were initially released to surface waters and outflow. This is not desirable if these metals are transported downstream. There were then differences between the measured elements in their response to conditions which progressively favored retention within sediments and subsequent removal from surface waters. The sediments soon became anoxic and the redox was low. The formation of metal sulfide precipitates and metal polysulfide complexes under anoxic conditions have been documented for: As, Cu, Fe, Mo, Zn, Co, Cr, Mn, and Ni [46,47,48]. Sorption to organic matter, clays, and oxide minerals would also be expected, especially for As and Cr [49,50] as well as the formation of metal selenide and elemental Se precipitates [51]. However, overall removal during this study was observed only for Sink elements (As and Se). In sharp contrast, Source elements (Co, Cr, Mg, Mn, Ni, S, and Sr) were continuously released to outflow. It is unlikely that biodegradation contributed significantly to these outward fluxes because most of the organic matter was removed from the wetland during the drying period. In addition, wetlands cannot be a permanent source of metals unless they are supplied from the atmosphere (e.g., Hg and Pb).

When sulfide-containing sediments are exposed to oxidizing conditions for long periods, previously bound metals are released and free sulfide is lost [39,52]. In the present study, total S was both removed from and released to surface waters resulting in overall net release. S speciation measurements were not part of the original study [32] and were not possible with the samples reported here. However, these data are consistent with S removal for sulfate reduction, as would be expected since sulfate reduction is considered a major if not predominant form of microbial respiration in coastal wetlands [53,54]. Release of sulfate is also expected following flooding of dried sulfidic sediments [22,55]. Importantly, the weekly mass flux rates for: As, Cu, Fe, Mo, Zn, Co, Mn and Ni increased and decreased in conjunction with removal rates for total S (*p* < 0.02), suggesting that the fluxes for these elements were at least partially the result of metal sulfide formation and oxidation.

During the months when the wetland was kept dry, periodic wet/dry cycles occurred from rain events which favored the accumulation of dissolved metals with Fe(III) and Mn(IV) oxides throughout the upper sediments. Dried sulfidic sediments that have undergone cyclic or short-term hydrologic inundation are known to be enriched in poorly-crystalline Fe(III) oxides and sorbed or co-precipitated trace metals [20,26,27]. Following re-flooding, the mobilization and prolonged release of most metals to porewaters, was likely controlled by the reductive dissolution of Fe(III) and Mn(IV) oxides, which are known to control metal release under reducing conditions [4,23,24,25].

In Fe-reducing sediment zones, the porewater concentrations of dissolved Fe(II) are typically elevated and are inversely related to dissolved sulfide concentrations [30,37,40]. The mean porewater Fe measurements made here (3.6 mg/L, Table 2) are similar to porewater measurements made in a local natural tidal wetland for dissolved Fe(II) (3.0 mg/L) which was typical of the region [56]. Wetland and lake studies typically find that when porewater Fe(II) concentrations are between 2.8 and 5.6 mg/L (50–100 uM), sulfide is less than 190 µg/L (6.0 uM) (e.g., [28,57,58,59]). Thus, the Fe porewater concentrations observed here are not only consistent with abundant reactive Fe(III) oxides but also low sulfide concentrations.

### 4.2. Metal(loid) Exchange between Sediments and Surface Waters

When the pool of available metals exceeds available sulfide, a thermodynamic equilibrium model put forth by Druschel et al. [60] predicts that less-soluble metal sulfides form before those that are more soluble. Under sulfate-limiting conditions Weber et al. [31] found that the relative order of sequestration for multiple metals followed the increasing solubility products for the respective metal sulfides, the thermodynamically-predicted ‘sulfide ladder’. In laboratory studies of temporarily flooded riparian floodplain soil, they determined that the amount of sulfide produced after 52 days was insufficient to sequester the entire pool of mobilized metals. Only the least soluble sulfide metals (Cu and Cd) were sequestered with sulfides, more-soluble Pb was partially sequestered, and the most soluble sulfide metals (Fe, Zn, Ni, and Mn) were released to porewaters [31]. In the present study, the mass flux rates for sulfide metal(loid)s also followed the increasing solubility products for the respective metal sulfides (Table 2) and the flux patterns were similar. Removal partially occurred for the least soluble sulfide metalloid, As (Sink), was marginal for more-soluble sulfide metals Cu, Fe, Mo and Zn (Zero-flux) and the most soluble sulfide metals Co, Mn, and Ni (Source) were continuously released to surface waters and outflow. Removal for As > Cu, Fe, Mo, Zn > Co, Mn, Ni is consistent with the thermodynamically-predicted sulfide ladder, suggesting that the extent to which these elements were released to or removed from surface waters was at least partially regulated by a limited supply of sulfide in porewaters. The differences between the three flux groups suggests that sulfide was consumed by the most insoluble sulfide metal(loid)s (Zero-flux and As).

The flux patterns observed here are interesting given that total S was high. In Weber et al. [31], metals were exchanged between solid phase and porewaters in freshwater microcosms where the pool of available metals exceeded available sulfate and sequestration patterns were attributed to limited sulfide production due to limited sulfate availability. In the present study, metal(loid)s were exchanged between upper sediments and surface waters in a dried/re-flooded coastal system where total S was several orders of magnitude greater than the concentrations of metals in porewaters, suggesting that available sulfate was much greater than the pool of available metal(loid)s. As discussed above, Fe(III) oxide enrichment is a common feature of dried sulfidic sediments. In addition, it is well established that sulfide is buffered to low levels within Fe-reducing zones. Therefore, we suggest that the pool of Fe(III) and Mn(IV) oxides here were sufficiently abundant to buffer sulfide to low concentrations and that available sulfide was insufficient to sequester the entire pool of mobile chalcophile elements. As a result, available sulfide was preferentially consumed by the least soluble metal(loid)s (As, Cu, Fe, Mo and Zn) and was insufficient to sequester the more-soluble Source elements (Co, Mn and Ni) which were released to surface waters and outflow.

Incorporation with Fe sulfides can also be an important sink for trace metals [46,61] in addition to the formation of monosulfides. FeS would be expected to be important under the study conditions and is known to effectively scavenge a wide range of metals [4,13]. Morse and Arakai [62] found that both adsorption and coprecipitation with FeS increased with decreasing sulfide solubility for divalent cations, which is consistent with the relative order of mass removal observed here for Zero-flux elements Cu, Fe, Mo and Zn > Source elements Co, Mn and Ni.

Research is needed to determine how trace metal/sulfide dynamics may be affected by Fe(III) and Mn(IV) oxide enrichment caused by drying. It would be important to determine how Fe(III) and Mn(IV) oxide abundance and reactivity are affected by drying duration as well as factors controlling Fe(III) and Mn(IV) oxide dissolution and persistence following re-flooding. Keene et al. [63] and Johnston et al. [20,21] found that pyritization was limited for Fe and trace metals within sediments that were highly enriched in reactive Fe(III) oxides 5 years after tidal inundation was restored to a salt marsh system that had been drained for 30 years. In an estuarine system historically impacted by Fe-rich runoff and following ~10 years of remedial freshwater flooding, Johnston et al. [28] found that high accumulations of Fe(III) oxides persisted which buffered sulfide to low levels, altered sulfidization processes and enhanced trace metal bio-availability, compared to a control site with low Fe(III) and higher sulfide. The short-term mobilization of metals immediately following drying/re-flooding was investigated by Karimian et al. [5] in an acid sulfate soil wetland re-flooded with freshwater. They found an initial release of acidity and metals (Al, Mn, Zn and As) for up to 7 days following re-flooding, followed by rapid sequestration of Fe, S, Zn and As; Fe(III) and sulfate reduction initiated within 4–8 weeks.

We propose then, that when the wetland was dried out, the initial release of metal(loid)s resulted from the oxidation of metal sulfides and other reduced forms (e.g., selenides) which had become sequestered during previous years of water treatment. Much of the released Fe and Mn were retained as Fe(III) and Mn(IV) oxides resulting in a vadose zone enriched in newly-formed metal sorbents. During wet/dry cycles, a significant portion of the released metal(loid)s were mobilized and accumulated with Fe(III) and Mn(IV)oxides throughout the upper sediments. Following re-flooding, the prolonged release of metal(loid)s to porewaters was controlled by the reductive dissolution of Fe(III) and Mn(IV) oxides, which remained incomplete. In addition, the enrichment of Fe(III) oxides buffered sulfide to low levels which were insufficient to sequester the entire pool of mobile metals. Consequently, metal(loid)s were ultimately released to surface waters or were sequestered with sulfides according to relative differences in their sulfide solubility. Sulfide was sufficient to deplete mobile elements from porewaters to the degree that removal from surface waters could occur only for the least soluble elements (Zero-flux and As). Chalcophile source metals (Co, Mn, and Ni) were continuously released to surface waters because they form the most soluble sulfides relative to the pool of metals that were present in porewaters.

Although the results for chalcophile elements are consistent with the hypothesis described above, results can hardly be conclusive due to the lack of supporting measurements that were available for the present work. Future research should include S speciation and measurements of metals associated with different Fe, S and organic fractions.

#### 4.2.1. Zero-Flux Elements

Patterns of mass removal and exchange between surface waters and sediments were distinctly different for Zero-flux elements. Periods of removal from inflow, especially during Period 2, offset release to outflow for Zero-flux elements, except for Fe (Figure 2). The weekly porewater concentrations for all Zero-flux elements did not significantly change over time, including during periods of sustained mass exchange, and were not correlated with surface water concentrations, except for Zn. This suggests that in addition to diffusion, the porewater concentrations for Ba, Cu, Fe, and Mo were regulated by other processes, such as solid-phase formation, and that the porewater concentrations of Zn were more dependent on surface water concentrations. One explanation for this dynamic is that the porewater concentrations for these elements were regulated by a continuous, but limited, supply of sulfide in porewaters. The fact that Zn was the most soluble Zero-flux element (by nearly 5 orders of magnitude), suggests that sulfide was preferentially consumed by the less-soluble Zero-flux elements (Cu, Fe, and Mo). In addition, a portion of these elements were likely complexed with dissolved organic ligands. Complexation with organic matter plays an important role in trace metal mobility and stability in wetlands [24,64] and can maintain metals in solution and reduce their activity. For example, Charriau et al. [65] found that porewaters consisting of 45–85% of Zn complexed with humic acids and 15–55% present as free Zn^2+^ accounted for porewater and sediment measurements at three riverine sites, while the overall low porewater concentrations were due to formation of Zn sulfides. Similarly, ElBishlawi et al. [66] reported that a large portion of Cu and Zn were complexed with dissolved organic carbon in near surface porewaters while the overall low porewater concentrations were due to sulfide formation at depth in two tidal marshes.

In addition, the Fe oxide plaques which form within the rhizosphere of wetland macrophytes have been shown to be important to the partitioning of a number of metal(loid)s in sediments including As, Cu, Fe, Mn, Zn, and possibly Sr [67,68]. Hansel et al. [67,68] estimated that 10% of As, 9% of Fe, and 5% of Zn sequestered by sediments were associated with plant roots in a wetland dominated by *T. latifolia* and *Phalaris arundinacea* (reed canarygrass). They observed maximum metal accumulation occurred in July and coincided with peak Fe plaque formation, seasonal development of emergent biomass, and peak photosynthesis. Therefore, accumulation with root plaques and plant uptake (discussed below) may have contributed to the removal of As, Zero-flux elements and Sr that occurred in this study, especially during Period 2 (24 July–14 August).

The water feeding the wetland consisted of treated wastewater, which was well within legal compliance limits. Therefore, the concentrations of the measured elements within the wetland should be less than would cause toxicity, but not necessarily less than would be limiting for growth, in the case of required elements. All Zero-flux elements are micronutrients, essential for the normal metabolic functioning of wetland biota, except for Ba. For example, Mo is required for nitrate reductase [69], and Cu, Zn, and Fe are cofactors required by proteins involved in photosynthesis [70,71]. Although Ba is not required for growth, its flux from surface waters has been shown to be controlled by seasonal biotic processes [72]. This flux is primarily due to the adsorption of barite to sinking plankton and organic matter. Note that Sr was also significantly removed from inflow during Period 2 (Figure 3B,C), and that Sr can substitute for Ca in biological processes [73].

Plant growth across the wetland was most rapid in the weeks just before and during Period 2. This time period is consistent with summer peaks in plant productivity and biological uptake processes reported from other natural and constructed wetland systems [74,75,76], and as discussed above for *T. latifolia* [67,68]. Therefore, it seems plausible that biological processes, such as plant and algal uptake, may have contributed to the removal of Zero-flux elements and Sr during Period 2. Further research is needed to confirm or deny this hypothesis.

#### 4.2.2. Sink Elements

The removal of Sink elements (As and Se) from surface waters was likely due to several mechanisms in addition to precipitation within sediments. Rates of mass removal were significantly lower during the second half of the study for both As and Se (Figure 4B,C). This would not be expected if removal was entirely due to sulfide or selenide precipitation because environmental conditions grew more reducing with time.

Arsenic removal rates averaged over 20% during the first half of the study, then decreased as flooding conditions continued, resulting in net release to outflow during Period 4. Interestingly, As porewater concentrations significantly decreased over time (*p* < 0.02) and remained significantly lower than surface water concentrations during Period 4 (Figure 4A), indicating that the release of As to surface waters occurred from the surficial sediments, which were located above the porewater measurements made here (1–11 cm below the sediment surface). In addition, since this significant release of As to outflow, apparently from surficial sediments, did not result in increased porewater concentrations suggests that a secondary mechanism, such as solid-phase formation or other mechanisms (discussed below) continued to deplete As from porewaters. This dynamic is consistent with laboratory studies which have shown that under oxic to sub-oxic conditions, As is controlled by adsorption/desorption with Fe minerals [50,77], in this case likely the reductive dissolution of newly-formed Fe(III) oxides in the surficial sediments [78]; while under reducing conditions As is controlled by precipitation with sulfides [35,48,79]. The release of As during Period 4 was likely due to the development of reducing conditions within the surficial sediments resulting from the >5.0 cm thick mat of plant material which had covered most of the sediment surface from plant dieback which was complete during Period 4 (9/18-10/9).

In addition, As may have also been released from wetland plant roots and plaques corresponding with seasonal dieback, as discussed above. High concentrations of dissolved organic carbon, which likely increased during the study, can also result in seasonally variable metal release in wetlands [24,64]. It is also possible that As was released to porewaters during Period 4, resulting in a reduced capacity of the sediments to sequester As from surface waters.

Therefore, the decreased As removal rates during the second half of the study were likely due to a combination of factors including destruction of and release from adsorption sites within surface sediments, plant dieback and depuration, and possibly other seasonal factors discussed below. Overall, these data suggest that a portion of mobile As (from inflow and sediment release) was temporarily sequestered with Fe minerals in the upper sediments, while the overall net removal from inflow was due to immobilization in deeper sediments. Partitioning studies over a year or more are needed to fully elucidate the significant fractions which contribute to As cycling in this system.

Se removal rates decreased somewhat as flooded conditions continued, from 75% during the summer months to 50% during fall (Figure 4B,C). Decreased temperatures may have affected sediment loading capacity (for both As and Se) by slowing the formation of microbially-mediated precipitates, sulfate reduction or other biological transformations. Previous mass balance estimates within this wetland could not fully account for the partitioning of Se between surface waters, plants, and sediments [34]. In a previous investigation, Hansen et al. [32] showed that biological volatilization resulted in significant loss of Se from this wetland. Se volatilization rates varied seasonally from various vegetated and non-vegetated sites and sometimes accounted for 10–30% of Se removal. The highest volatilization rates were measured in the early spring and summer months when Se removal rates were highest, suggesting that seasonally-declining volatilization rates may have contributed to the lower Se mass removal rates during the second half of the study. Similar strong seasonal fluctuations in the production of volatile As species have been documented in other aquatic systems [80,81,82] and may have contributed to the mass fluxes observed here.

## 5. Conclusions

In response to drying/re-flooding, different elements exhibited positive, negative, and zero overall mass fluxes between surface waters and sediments which can be attributed to different biotic and abiotic factors. Initially, Source (Co, Cr, Mg, Mn, Ni, S, and Sr) and Zero-flux elements (Ba, Cu, Mo, and Zn) were mobilized and released to surface waters and outflow. As re-flooding continued, release was offset by removal for Zero-flux and Sink (As and Se) elements, whereas Source elements were continuously released. Relative differences between mass removal rates for As > Cu, Fe, Mo, Zn > Co, Mn, Ni followed the increasing solubility products for the respective metal sulfides, the thermodynamically-predicted sulfide ladder, suggesting that mass exchange between pore- and surface waters for these elements was at least partially regulated by a limited supply of sulfide in porewaters. The results suggest that less-soluble sulfide metal(loid)s may be immobilized prior to more-soluble metal(loid)s following drying/re-flooding in Fe- and S-rich systems with multiple metal contaminants. In addition, seasonal biological processes such as plant and algal uptake may have contributed to the removal of Zero-flux elements (except for Fe) and Sr that occurred during Period 2. Biological volatilization was important for Se, however, volatilization rates may have declined seasonally resulting in lower mass removal rates for Se, and possibly As, during the second half of the study. Arsenic mass flux patterns over time were also consistent with previously reported patterns of retention with and release from Fe(III) oxides under oxic-suboxic conditions, while sequestering with sulfides at depth. Further investigation is warranted to understand the effects of drying/re-flooding on trace metal(loid) mobilization and Fe-S dynamics in treatment wetlands and other similar systems such as BMP basins, stormwater impoundments, low-lying coastal areas subject to sea level rise and re-claimed natural wetlands with metal contamination. Due to the highly seasonal nature of wetlands, biological and geochemical factors must be studied over year or more in order to assess trace element dynamics, or to optimize annual removal patterns.

Clearly, treatment wetlands and BMP detention basins should not be put into full operation following drying/re-flooding if downstream releases are involved. We suggest that temporary ponding or other similar strategies should be used to re-establish anoxic conditions necessary for metal immobilization prior to discharging waters to the environment. This would minimize release of oxidized metals from the system and would facilitate their re-sequestration within sediments, thereby limiting exposure to biota. Design of the process train for treatment wetlands with two parallel tracks is desirable for maintenance and could become more common for BMP detention basins. A multi-train design allows water treatment to continue in one treatment train while one is dried out and re-flooded.

Treatment wetlands are increasingly being used to remove large and small amounts of metal contamination from wastewaters. However, wetlands are often dried out or operated on a seasonal (wet/dry) basis. This study indicates that this mode of operation may substantially reduce their effectiveness as metal sinks. The mass flux data presented here do not apply to systems which remain anoxic, or continuously-flooded wetlands.

## Figures and Tables

**Table 1:** The 16 week mean values (±standard deviation) for pH, electrical conductivity (EC), and redox potential (Eh) measured in wetland inlet, outlet (*n* = 16), and porewaters (*n* = 60–80).

	pH	EC (µS cm^−1^)	Eh (mV)
**Inlet**	7.34 ± 0.22	3600 ± 700	281 ± 251
**Outlet**	7.31 ± 0.19	4100 ± 400	288 ± 241
**Porewater**	7.18 ± 0.20	6200 ± 2500	222 ± 294

**Table 2:** Pooled (16 week) mean values for total metal, As, Se and S concentrations in inlet, outlet and porewaters (µg or mg */L ± standard deviation), removal rate (g or kg **/day ± standard error) and percent removed from inlow (±standard error) for Sink, Zero-flux and Source elements measured during the study period. Value superscripts (^a–c^) indicate statistically significant differences (ANOVA, *p* < 0.05, Tukey–Kramer). (*n* = 16 for inlet and outlet values; *n* = 60–80 for porewater values). Selected solubility product constants for sulfide minerals corresponding to the measured metal(loid)s.

Elements	Inlet Concentration (µg or mg */L)	Outlet Concentration (µg or mg */L)	Porewater Concentration (µg or mg */L)	Rate of Mass Removal (g or kg **/day)	Percent Removed from Inflow	Log K′_sp_ of Sulfide Mineral ^§^
**Sink**						
**As**	**36 ± 15 ^a^**	**28 ± 9 ^a^**	**18 ± 8 ^b^**	**30 ± 15**	**10 ± 6**	**−64.3 ^¶^**
**Se**	**23 ± 6 ^a^**	**7 ± 4 ^b^**	**6 ± 3 ^b^**	**96 ± 10**	**65 ± 4**	**n.a. ^#^**
**Zero-Flux**						
**Ba**	**50 ± 12 ^a^**	**49 ± 15 ^a^**	**70 ± 25 ^a^**	**−29 ± 35**	**−10 ± 11**	**n.a.**
**Cu**	**31 ± 38 ^a^**	**26 ± 20 ^a^**	**21 ± 14 ^a^**	**7 ± 89**	**−62 ± 60**	**−23.2**
**Fe**	0.14 ± 0.08 ^a,^*	0.13 ± 0.1 ^a,^*	3.57 ± 2.15 ^b,^*	**−32 ± 177**	**−22 ± 25**	**−4.5_mac_^†^ −19.0_py_**
**Mo**	**65 ± 10 ^a^**	**61 ± 18 ^a^**	**77 ± 68 ^a^**	**−16 ± 60**	**−6 ± 13**	**−72.8 ^‡^**
**Zn**	**274 ± 80 ^a^**	**250 ± 75 ^a^**	**210 ± 91 ^a^**	**85 ± 111**	**−1 ± 8**	**−12.4**
**Source**						
**Co**	**4 ± 1 ^a^**	**6 ± 2 ^a^**	**22 ± 13 ^b^**	**−17 ± 5**	**−82 ± 19**	**−8.3**
**Cr**	**11 ± 10 ^a^**	**15 ± 15 ^a,b^**	**23 ± 13 ^b^**	**−29 ± 21**	**−97 ± 45**	**n.d.**
**Mg**	24 ± 5 ^a,^*	30 ± 6 ^a,^*	88 ± 32 ^b,^*	−49.1 ± 18.2 **	**−38 ± 11**	**n.a.**
**Mn**	**51 ± 37 ^a^**	**227 ± 212 ^b^**	**448 ± 189 ^c^**	**−1094 ± 260**	**−432 ± 94**	**−0.7**
**Ni**	**58 ± 35 ^a^**	**73 ± 24 ^a^**	**114 ± 53 ^b^**	**−143 ± 112**	**−83 ± 38**	**−6.5**
**S**	2680 ± 920 ^a,^*	2910 ± 1030 ^a,^*	2510 ± 870 ^a,^*	−2640 ± 1650 **	**−20 ± 9**	**n.a.**
**Sr**	0.61 ± 0.14 ^a,^*	0.68 ± 0.11 ^a,^*	1.33 ± 0.36 ^b,^*	**−693 ± 333**	**−24 ± 9**	**n.a.**

^§^ Selected solubility products for orpiment (As_2_S_3_), covellite (CuS), mackinawite (FeS), pyrite (FeS_2_), molybdenite (MoS_2_), sphalerite (ZnS), cobalt sulfide (CoS), albandite (MnS) and millerite (NiS), respectively. Data are from the Minteq.v3 database [41]. Solubility products were adjusted for ionic strength (I = 0.1) using porewater conductivity data [42] and the Davies equation [43]. n.a. = not applicable, n.d. = no data. ^¶^ The solubility of orpiment (As_2_S_3_) would be expected to increase under the pH conditions of this study [44]. ^#^ Metal-selenides should form before corresponding metal sulfides because they are thermodynamically less soluble (e.g., Log Ksp = −33.1 (CuSe); −11 (FeSe); −14.4 (ZnSe); −16.2 (CoSe); −3.5 (MnSe); −17.7; (NiSe)) [41]. ^†^ Mackinawite forms before pyrite under the study conditions due to faster reaction kinetics [13]. ^‡^ The formation of MoS_2_ under wetland conditions is thought to be kinetically limited (requiring temperatures > 200 °C) and is likely mediated by sulfate-reducing bacteria [45].

## Data Availability

The data presented in this study are available in online supplement.

## Supplementary Materials

The following are available online at https://www.mdpi.com/article/10.3390/biology11020188/s1, Data S1: Figure and table data.
